# A Novel N-Sulfonylamidine-Based Derivative Inhibits Proliferation, Migration, and Invasion in Human Colorectal Cancer Cells by Suppressing Wnt/β-Catenin Signaling Pathway

**DOI:** 10.3390/pharmaceutics13050651

**Published:** 2021-05-03

**Authors:** Xingming Zhao, Zhuo Han, Jiahui Ma, Shiqing Jiang, Xia Li

**Affiliations:** 1 Marine College, Shandong University, Weihai 264209, China; zhaoxingming@mail.sdu.edu.cn (X.Z.); hanzhuo1013@gmail.com (Z.H.); sdumjh@gmail.com (J.M.); zoemh48@gmail.com (S.J.); 2 School of Pharmaceutical Sciences, Shandong University, Jinan 250012, China

**Keywords:** colorectal cancer, HCT-116 cells, 26ag, EMT, Wnt/β-catenin

## Abstract

Wnt signaling has been implicated in the development and metastasis of colorectal cancer (CRC), as well as poorer outcomes. Thus, targeting the Wnt/β-catenin signaling pathway is expected to be a promising treatment option for the therapy of advanced metastatic CRC. A new N-sulfonylamidine derivative (26ag) has been confirmed to suppress the growth of tumor cells by inhibiting C-met, showing strong anti-cancer activity. In this paper, we test the effectiveness of 26ag in suppressing CRC cell proliferation, invasion, and migration. In this regard, 26ag decreased the mRNA and protein expressions of important hallmarks associated with epithelial to mesenchymal transition (EMT). Furthermore, we provide evidence that β-catenin-dependent signaling is involved in 26ag-induced Wnt/β-catenin pathway effects in CRC, using in vitro cell culture and computer docking models. Our study indicates that inhibition of Wnt/β-catenin by a novel compound, 26ag, demonstrates possibility for drug development in the therapy of CRC.

## 1. Introduction

Colorectal cancer (CRC) is the second primary cause of cancer deaths in the United States and one of the most universal gastrointestinal malignancies in the world [1,2]. Over the past few decades, morbidity and mortality associated with CRC have steadily risen [3]. According to American Cancer Society research, in the United States, approximately 104,270 people will be diagnosed with CRC in 2021, while more than 52,980 people will die from the disease [4]. At present, standard treatment schedules adopted for CRC include surgical resection with radiotherapy/chemotherapy, for example, endoscopic or segmental resection is used to treat malignant colorectal polyps, while cancers with high metastatic potential are surgically removed and treated with adjuvant chemotherapy, immunotherapy, or radiation therapy to inhibit metastasis to tissues near the cancerous focus [5,6,7]. According to previous studies, even if tumorous tissue is removed prior to tumor cell metastasis, nearly 50% of CRC patients have tumor recurrence, while the effects of chemotherapy are significantly reduced on advanced malignant colorectal tissue [8,9]. Despite numerous efforts, the progress in new treatments for CRC has been pessimistic. Therefore, it is necessary to search for new drugs for the treatment of CRC.

The Wnt/β-catenin signaling pathway is a crucial regulator of the equilibrium of normal intestinal stem cells (ISCs) [10]. Abnormal activation of Wnt/β-catenin signaling has been implicated in all kinds of human cancers, including CRC, lung cancer, pancreatic cancer, and breast cancer [11,12,13,14,15]. The Wnt/β-catenin signaling pathway is abnormally activated in cancer cells, which has mainly been attributed to somatic mutations in the Wnt signaling component, adenomatous polyposis coli (APC), as well as β-catenin gene mutations that occur in approximately 90% of CRC patients, that disrupt the Wnt gradient and cause ISCs to proliferate along the crypt–villus axis [16,17,18]. In the process of Wnt/β-catenin signal activation, β-catenin breaks away from the APC/Axin/GSK-3β/β-catenin degradation complex, collects in the cell substance, and then migrates to the nucleus, where it interacts with the nuclear transcription factor LEF1/TCF (usually combined with TCF-4) that activates the transcription of a series of Wnt downstream target genes (such as MMP7, c-jun, cyclinD1, and c-myc), which triggers cell proliferation and malignant transformation [19]. Additionally, the Wnt/β-catenin signaling pathway performs essential functions in the activation and maintenance of CRC, and activation of this pathway is a sign of poor prognosis in CRC patients [20,21]. When the Wnt/β-catenin signaling pathway is abnormally activated, cells exhibit abnormal proliferation, invasion, and metastatic capabilities. Thus, decreasing β-catenin expression in cancer cells has become a key target for reducing abnormal proliferation and migration of tumor cells.

Presently, the main medicines used for the treatment of CRC are 5-fluorouracil and oxaliplatin [22]. The major side effects of oxaliplatin are a rapid reduction of platelets in the body, elevated temperatures, kidney failure, hemolytic anemia, shivering, and hemorrhage [23]. Meanwhile, the main issue with 5-fluorouracil (5-FU) treatment is that in most patients, the treatment cannot completely remove all tumor cells, which results in recurrence following 5-FU treatment and, ultimately, a poor prognosis [24]. Therefore, in the process of drug development, full consideration should be given to reducing drug toxicity and improving drug efficacy. Targeted therapy using small molecule compounds can target specific mutations in cancer cells and reduce damage to normal cells in the body. Currently, because amidines and sulfonates play a fundamental role in the selection of a variety of low-toxicity anti-tumor drugs, they continue to attract attention [25,26]. Sulfonylamidine has the potential to become an effective anti-tumor drug linker [27]. Our previous report demonstrates that N′-((3,4-dichlorobenzyl)sulfonyl)-N-(3-fluoro-4-((6-methoxy-7-(3-morpholinopropoxy)quinolin-4-yl)oxy)phenyl)-3-methoxypropanamidine(26ag), a novel N-sulfonylamidine-based derivative, has excellent antitumor effects in vitro [28].

In this study, in order to further explore the effect of 26ag in CRC cells and assess the potential molecular mechanism of 26ag in HCT-116 cells, we test the effectiveness of 26ag in suppressing CRC cell through related experiments, which may provide a novel strategy into the Wnt/β-catenin signaling pathways on CRC therapy.

## 2. Materials and Methods

### 2.1. Chemicals and Reagents

Compound 26ag (purity > 98%) was designed and synthesized in the School of Marine Science and Technology, Harbin Institute of Technology, Weihai, China [28]. All antibodies were purchased from Cell Signaling Technology (CST, Inc., Beverly, MA, USA). Compound 26ag was dissolved in dimethylsulfoxide (DMSO) (Sigma-Aldrich Corp., St. Louis, MO, USA) to obtain a stock solution which was then diluted in serum-free medium.

### 2.2. Cell Lines and Cell Culture

The human CRC cell lines, HCT-116, LoVo, and SW620, as well as the normal human liver cell line, L-O2, were purchased from the Shanghai Institute for Biological Sciences (SIBS, Shanghai, China). CRC cells (HCT-116, SW620) were cultured in RPMI 1640 Medium (Livning Biotechnology Co., Ltd., Beijing, China), LoVo and L-O2 cells were cultured in DMEM/F-12(Livning Biotechnology Co., Ltd., Beijing, China) and DMEM/high glucose (Livning Biotechnology Co., Ltd., Beijing, China) containing 10% fetal bovine serum (FBS). Cells were incubated at 37 °C in a dampened incubator with 5% CO_2_.

### 2.3. Cell Proliferation Assay

We adopted the MTT method to assess the inhibitory effect of 26ag on cell proliferation. Human HCT-116, SW620, LoVo, and L-O2 cells were seeded in 96-well plates (1 × 10^4^ cells/well). The next day, we treated the cells with increasing concentrations of 26ag, from 0 to 20 μmol/L. At the specified time points (24, 48, and 72 h), 15 μL of 5 g/L MTT (Sigma-Aldrich Corp., St. Louis, MO, USA) was added to the each well and left for 4 h. Then, 150 μL DMSO was added to each hole, and a microplate reader was used to detect the absorbance at 570 nm. The data were presented as the percentages of cell viability and the IC_50_ value was calculated. Each sample was measured three times separately.

### 2.4. DAPI Staining

HCT-116 cells were seeded in a 24-well plate containing a 12 mm round cover glass for culture. The cell density was about 3 × 10^4^/well. After the cells adhered to the wall, the cells were treated with 26ag at various concentrations. After 24 h of treatment, the cells were washed with PBS, fixed with fixative, and finally stained with 4 μg/mL DAPI (Beyotime Biotech, Shanghai, China). Then, the processed coverslip was placed under a fluorescence microscope for observation.

### 2.5. Flow Cytometry Analysis of Apoptosis

The Annexin V-FITC cell apoptosis detection kit (Beyotime Biotech, Shanghai, China) was employed to stain HCT-116 cells treated with 26ag, which were then analyzed by flow cytometry. The HCT-116 cells were inoculated in a 6-well plate (5 × 10^4^/well) and then treated with 26ag. The apoptosis rate was measured with a flow cytometer (Becton Dickinson FACScan, City, CA, USA). Each sample was measured three times separately.

### 2.6. Mitochondrial Membrane Potential Assay

The mitochondrial membrane potential was measured using the Mitochondrial Membrane Potential Measurement Kit with JC-1 (Beyotime Biotech, Shanghai, China), and the steps were performed according to the manufacturer’s instruction. In short, an equal number of HCT-116 cells were inoculated in a 6-well plate and treated with various doses of 26ag for 24 h. The cells were treated with JC-1 at 37 °C in the dark for 30 min, after which a fluorescence microscope was used for observation.

### 2.7. Transwell Assay

Cells, with a density of 5 × 10^4^ per well, were inoculated in the upper chamber, where 50 μL Matrigel was dissolved in a serum-free medium insert. The lower chamber contained 600 μL of 20% FBS medium. After 24 h of 26ag treatment at various doses, cells were fixed with formaldehyde for 15 min and then dyed with 1% crystal violet for 40 min. Pictures were taken under the microscope after washing the migrated cells three times, and 5 random fields were selected for quantification. For the migration experiment, the steps were performed as detailed for the above method except that Matrigel was not added to the chamber.

### 2.8. Wound-Healing Assay

To measure the capability of cells to migrate in vitro, we adopted the wound-healing method. The HCT-116 cells were inoculated in a 6-well plate with a density of 1×10^5^ cells/well. Once the cell cultures had reached approximately 90% confluence, a micropipette tip was used to vertically scrape the middle of each well. Then, the cells were treated with different doses of 26ag in serum-free growth medium for 24 h. Changes in the width of the scratch were observed under the microscope and documented for each group. Each sample was measured three times separately.

### 2.9. Real-Time PCR Analysis

Human HCT-116 cells were treated with varying doses of 26ag for 24 h. Total RNA was extracted from the cells using the RNAeasy kit (Sparkjade Biotec Co., Ltd., Shandong, China) according to the manufacturer’s instructions. The RNA was transformed into cDNA through the SPARK script II RTPlus kit (Sparkjade Biotec Co., Ltd., Shandong, China). The SPARK script II SYBR Green qRT-PCR Kit (Sparkjade Biotec Co., Ltd., Shandong, China) was used to measure the expression level of related genes. The 2^−ΔΔCT^ method was used to analyze the RT-qPCR data. Each sample was evaluated three times separately. The primer sequences used are displayed in Table 1.

### 2.10. Immunofluorescence Staining

HCT-116 cells were inoculated on a 24-well plate equipped with a 12 mm round cover glass for culture at a cell density of 5 × 10^3^ cells/well. Then, the cells were treated with various doses of 26ag for 24 h and then washed and fixed with 4% paraformaldehyde. Then, the cells were washed with cold PBS, permeabilized in 0.1% Triton X-100 for 20 min, washed again, and then incubated with 5% BSA for 30 min to block non-specific binding. Next, the preparation was incubated with β-catenin or GSK-3β antibody for 12 h at 4 °C and then with FITC goat anti-rabbit secondary antibody for 40 min. After being washed, the cells were stained with DAPI for 10 min. Finally, a fluorescence microscope was used to detect and take fluorescence images.

### 2.11. Western Blot Analysis

The HCT-116 cells treated with varying doses of 26ag were lysed with RIPA buffer (Beyotime Biotech, Shanghai, China) to obtain total protein, after which the BCA kit was used to quantify the obtained protein. Polyacrylamide gel electrophoresis was used for Western blot analysis and the detection of related protein expression, including GAPDH, Bcl-2, Bax, cleaved Caspase-3, slug, E-cadherin, GSK-3β, Vimentin, snail, β-catenin, N-cadherin, and TCF4. The proteins were transferred to a PVDF membrane that was then incubated in 5% skim milk for 1h to block non-specific protein binding. After washing with TBS-T buffer, the PVDF membrane was incubated in the corresponding primary antibody for 12 h at 4 °C. Next, the membrane was incubated with anti-mouse IgG or anti-rabbit IgG secondary antibody (CST, Inc., Beverly, MA, USA) for 1 h. The protein on the membrane was visualized using the enhanced ECL kit (ECL^®^, Amersham Biosciences, Little Chalfont, UK).

### 2.12. Docking Studies

Molecular docking research was performed using the related software, AutoDockTools-1.5.6. The β-catenin3D structure (PDB ID: 1JDH, 1.9 A resolution) was obtained in the PDB. In docking analysis, the conformation that has the lowest binding energy is selected as the most suitable for drugs and ligands. The docking method used in the docking analysis was the Lamarckian genetic algorithm.

### 2.13. Statistical Analysis

All data are shown as means ± SEM. One-way analysis of variance (ANOVA) was used to compare all data multiple times. Each experiment was repeated at least three times. One-way ANOVA and GraphPad Prism8.0 software (GraphPad, San Diego, CA, USA) were used for statistical analysis. The level of significance was *p* < 0.05.

## 3. Results

### 3.1. 26ag Inhibits Colorectal Cancer Cell Viability and Induces Apoptosis

**Figure 1:**
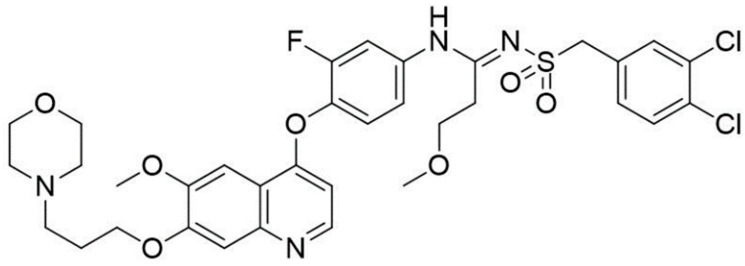
Chemical structure of 26ag.

**Figure 2:**
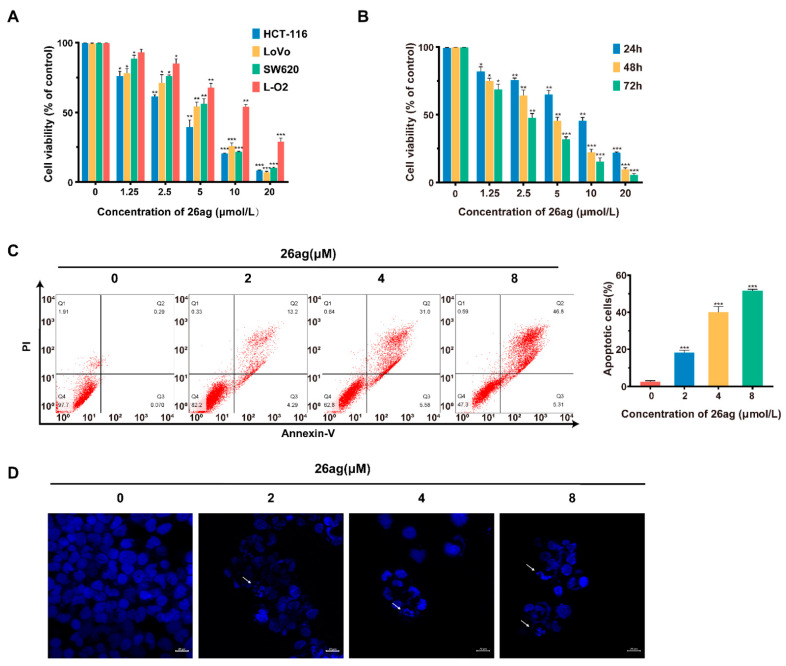
26ag suppresses proliferation and induces apoptosis in CRC. (**A**) 26ag treatment suppressed the proliferation of SW620, HCT-116, LoVo, and L-O2 cells, as shown by MTT assay; (**B**) 26ag time-dependently inhibits the proliferation of HCT-116 cells; (**C**) Flow cytometry quantitative detection of 26ag-induced HCT-116 cell apoptosis; (**D**) Fluorescence micrographs of HCT-116 cells with DAPI staining. Scale bars = 20 μm. * *p* < 0.05, ** *p* < 0.01, *** *p* < 0.001.

Firstly, in order to evaluate the cytotoxicity of 26ag (Figure 1), we performed MTT experiments on human CRC cells (HCT-116, LoVo, SW620) and normal human liver cells (L-O2) to evaluate the effects of 26ag on cell proliferation. We found that the IC_50_ values of 26ag on L-O2, SW620, LoVo, and HCT-116 cells were 10.51 ± 0.39 μmol/L, 5.45 ± 0.39 μmol/L, 4.90 ± 0.17 μmol/L, and 3.67 ± 0.19 μmol/L, respectively, when treated for 48 h. The data obtained show that 26ag inhibits CRC cells in a dose-dependent manner and is less cytotoxic to the non-cancerous L-O2 cells than to cancer cells (Figure 2A). Furthermore, 26ag time-dependently suppressed the proliferation of HCT-116 cells (Figure 2B). Since 26ag demonstrated the highest anti-proliferative capabilities on HCT-116 cells, we selected this cell line for further study. Next, we used flow cytometry to analyze 26ag-induced apoptosis and found that as the concentration of 26ag gradually increased, the content of apoptotic cells gradually increased (Figure 2C). In addition, adopting DAPI staining to explore the morphological changes of 26ag on HCT-116 cells in apoptosis, we found that the treated HCT-116 cells showed obvious nuclear condensation and the appearance of apoptotic bodies (Figure 2D).

**Figure 3:**
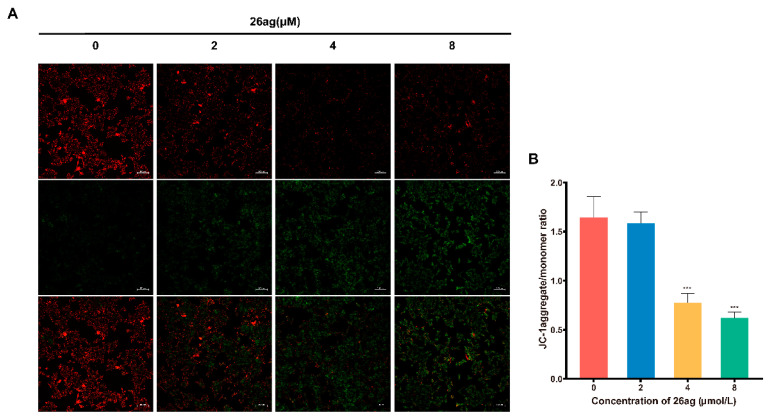
26ag decreased mitochondrial membrane potential (ΔΨm) of HCT-116 cells. (**A**) HCT-116 cells were treated with various concentrations of 26ag for 24 h and then treated with JC-1 probe and observed under a fluorescence microscope. The representative images for each condition are shown. Scale bars = 100 μm. Red fluorescence represents high ΔΨm forming aggregates, green fluorescence represents low ΔΨm forming monomers. (**B**) Analysis of the JC-1 aggregate/monomer ratio. *** *p* < 0.001.

Decreases in mitochondrial membrane potential (ΔΨm) are used as an indicator of the initial and irreversible stages of apoptosis [29]; the JC-1 fluorescent probe is employed to detect the ΔΨm. After entering non-apoptotic cells, JC-1 forms red aggregates in the matrix of mitochondria; conversely, green fluorescent monomers form in the early stage of apoptosis. With the help of the JC-1 probe, we found that as the dose of 26ag increased, the red fluorescence gradually decreased, while the green fluorescence gradually increased (Figure 3A). Based on these observations, we concluded that as the concentration of 26ag increases, ΔΨm decreases correspondingly (the aggregate/monomer fluorescence ratio decreases) (Figure 3B).

### 3.2. Effects of 26ag on the Expression of Apoptotic Proteins and mRNA in HCT-116 Cells

**Figure 4:**
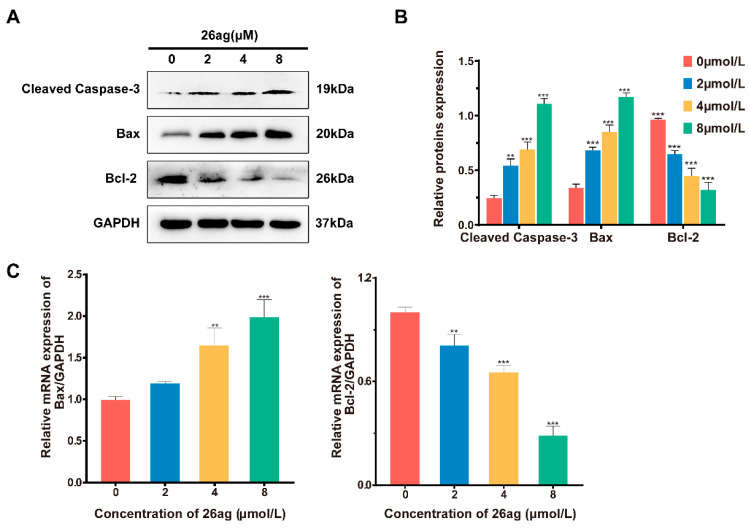
Trends of 26ag on Bcl-2, Bax, and cleaved caspase-3 expression in HCT-116 cells. (**A**,**B**) Western blot was used to detect the levels of Bcl-2, Bax, and cleaved caspase-3; (**C**) RT-PCR analysis of mRNA expression of Bax and Bcl-2 relative to GAPDH. ** *p* < 0.01, *** *p* < 0.001.

In the process of cell apoptosis, Bax, Bcl-2, and caspase-3 play a crucial role. Therefore, we analyzed changes in the expression of Bax, Bcl-2, and cleaved caspase-3 in HCT-116 cells treated with 26ag by Western blotting. The experimental analyses show that 26ag is capable of significantly increasing the protein expression of Bax and cleaved caspase-3 in HCT-116 cells while decreasing the expression of Bcl-2 (Figure 4A,B). We also analyzed the changes in Bax and Bcl-2 mRNA expression levels in HCT-116 cells treated with 26ag using RT-PCR (Figure 4C). We found that the addition of 26ag significantly reduced Bcl-2 mRNA levels and up-regulated the levels of Bax when compared with the control group. Therefore, the obtained results indicate that 26ag dose-dependently induces apoptosis of HCT-116 cells.

### 3.3. 26ag Suppress Migration and Invasion by HCT-116 Cells

**Figure 5:**
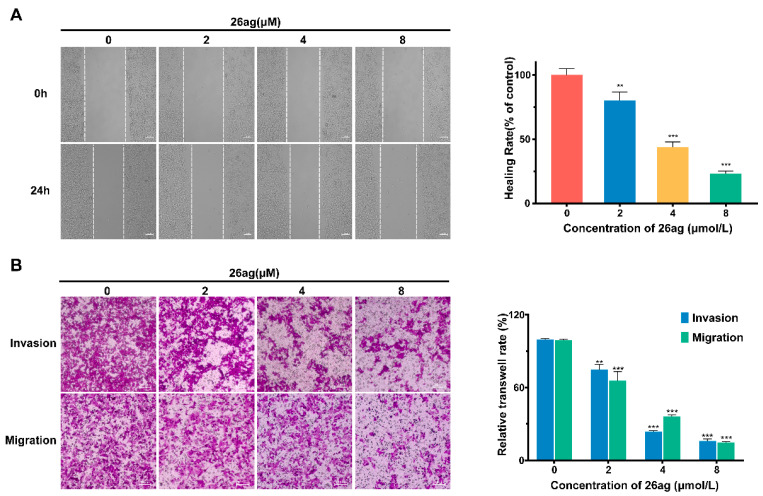
26ag suppress the migration and invasion ability of HCT-116 cells. (**A**) 26ag inhibits the migration capability of HCT-116 cells through the wound-healing experiment. Left: representative images for each condition are shown. Scale bars = 100 μm. Right: analyses of healing rate. (**B**) 26ag inhibits the invasion and migration ability of HCT-116 cells through the transwell assay. Left: representative images for each condition are shown. Scale bars = 100 μm. Right: quantification of the relative transwell rate. ** *p* < 0.01, *** *p* < 0.001.

Invasion ability and migration potential are crucial factors influencing the metastasis of malignant CRC. Therefore, we verified the ability of 26ag to inhibit HCT-116 cell invasion and metastasis through wound-healing analysis experiments and transwell assay. According to the results of the wound-healing experiment, 26ag is capable of greatly suppressing the migration capability of HCT-16 cells in vitro (Figure 5A). The healing rate of the group treated with the highest dose of 26ag (8.0 μM) was less than 8%, compared to about 30% in the control group. At the same time, the transwell assay showed that 26ag inhibits the invasion and metastasis of CRC cells (Figure 5B). As the dose of 26ag was incrementally increased, the invasion and metastasis of the cells decreased correspondingly.

### 3.4. Results of 26ag on the Levels of EMT-Associated Proteins and mRNA in HCT-116 Cells

**Figure 6:**
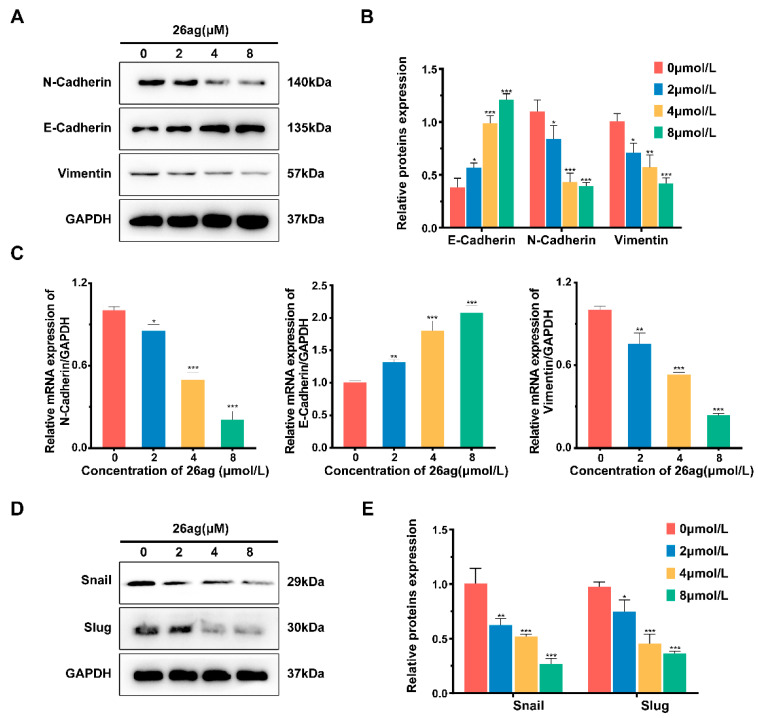
Trends of 26ag on EMT protein and mRNA expression in HCT-116 cells. (**A**,**B**) Western blot analysis was used to analyze the levels of E-cadherin, N-cadherin, and vimentin; (**C**) RT-PCR was used to detect the mRNA levels of E-cadherin, N-cadherin, and vimentin relative to GAPDH; (**D**,**E**) Western blot was used to analyze the levels of Snail and Slug protein expression. * *p* < 0.05, ** *p* < 0.01, *** *p* < 0.001.

Western blot analysis was used to evaluate the effects of 26ag on inhibiting epithelial to mesenchymal transition (EMT) of human HCT-116 cells. As illustrated in Figure 6A,B, after HCT-116 cells were treated with various doses of 26ag for 24 h, the levels of N-cadherin and Vimentin were significantly decreased, while the expression of E-cadherin was increased. Subsequently, we adopted RT-PCR to evaluate changes in the expression of relevant markers at the RNA level. The results of RT-PCR were consistent with the results of Western blotting; i.e., E-cadherin levels were up-regulated, while the levels of N-cadherin and Vimentin were down-regulated (Figure 6C). According to previous studies, the EMT process requires an extremely complex transcription factor regulation system. Snail family transcription repressor 1 (Snail) and Snail family transcription repressor 2 (Slug), being inhibitors of E-cadherin, are also inducers of the EMT process [30,31]. Western blot analysis of 26ag-treated HCT-116 cells found that the levels of Snail and Slug were significantly decreased (Figure 6D,E). In summary, 26ag dose-dependently inhibits the EMT process of CRC cells in a significant way.

### 3.5. 26ag Inhibits the Wnt/β-Catenin Signaling Pathway

**Figure 7:**
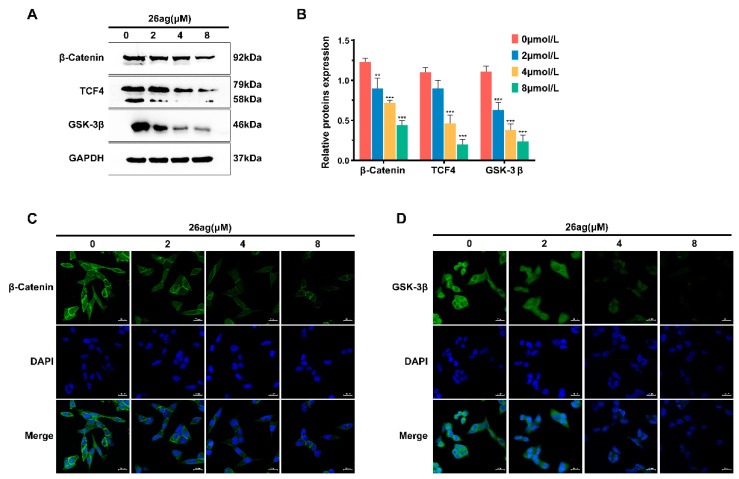
The effect of 26ag on the Wnt/β-catenin pathway in HCT-116 cells. (**A**,**B**) Western blot was used to analyze the levels of β-catenin, GSK-3β, and TCF4 in HCT-116 cells treated with 26ag. (**C**) Immunofluorescence staining was adopted to explore the expression of β-catenin. Representative images for each condition are shown, β-catenin (green) and DAPI (blue). Scale bars = 20 μm. (**D**) Immunofluorescence staining was adopted to explore the expression of GSK-3β. Representative images for each condition are shown, GSK-3β (green) and DAPI (blue). Scale bars = 20 μm. ** *p* < 0.01, *** *p* < 0.001.

Former research has shown that the Wnt/β-catenin signaling pathway is a classic signaling pathway that induces EMT and cell apoptosis. Combined with the previous experimental results, we considered the idea that 26ag causes CRC cell apoptosis and inhibits tumor cell growth and migration through the wnt/β-catenin signaling pathway. For the study, we treated HCT-116 cells with 26ag and detected the protein expression level of β-catenin, GSK-3β, and TCF4. As illustrated in Figure 7A,B, after HCT-116 cells were treated with various doses of 26ag for 24 h, the expression levels of β-catenin, GSK-3β, and TCF4 were significantly down-regulated relative to the control group. Interestingly, through immunofluorescence staining, we found that the various concentrations of 26ag not only suppressed β-catenin expression but high concentrations of 26ag also inhibited the transport of β-catenin to the nucleus (Figure 7C). As a component of the APC/Axin/GSK-3β complex, GSK-3β performs a vital role in the Wnt/β-catenin signal transduction pathway. Immunofluorescence staining of GSK-3β showed similar reductions after 26ag treatment (Figure 7D). In summary, according to the above experimental results, 26ag inhibits the proliferation, vitality, invasive ability, and metastatic capacity of CRC cells, and it induces cell apoptosis, most likely through the Wnt/β-catenin pathway.

### 3.6. Molecular Docking Analysis of 26ag against β-Catenin

**Figure 8:**
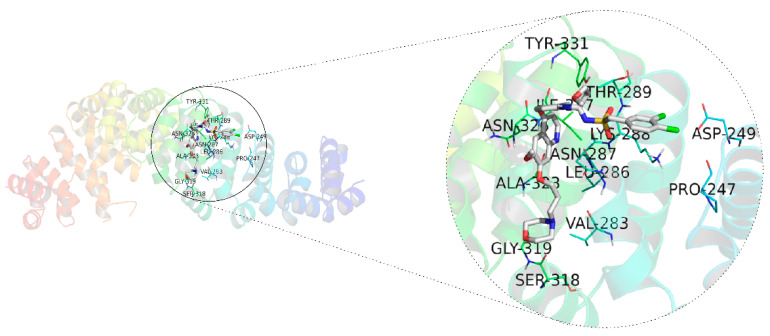
AutoDockTools software was employed to obtain the optimal docking model of 26ag to the β-catenin protein, and the resulting image is a panoramic view of the optimal binding method. The lowest binding energy configuration is −3.05 kcal/mol.

In order to further determine whether 26ag suppresses the Wnt/β-catenin signaling pathway by interacting with β-catenin, as well as to predict its binding mode at the active site of β-catenin, we employed the AutoDockTools-1.5.6 software package to conduct a molecular docking simulation study. During software simulation of molecular docking, the conformation that has the lowest binding energy is selected as the most suitable binding site for drugs and ligands. According to the simulated docking results, the optimal binding mode of compound 26ag and β-catenin is shown in Figure 8. The results of the docking analysis showed that the lowest binding energy of 26ag was -3.05 kcal/mol and the inhibition constant (Ki) was 5.77 mM. Additionally, we found that 26ag is in a favorable position in the β-catenin domain and binds through strong interaction. According to the results of molecular docking, we speculate that 26ag may inhibit the Wnt/β-catenin signaling pathway by binding to β-catenin.

## 4. Discussion

Despite the constant drive for advances in new therapeutic strategies for CRC treatment, neoplasms with a high rate of malignancy and metastasis are still considered incurable [32]. The development of drugs with outstanding clinical activity and low toxicity has gradually attracted people’s attention. Using rational screening of synthetic N-sulfonylamidine derivatives, we identified 26ag suppressed CRC cell proliferation, invasion, and migration while displaying low toxicity against hepatic cells; therefore, there may be a therapeutic window of 26ag worthy of further investigation. It appears that 26ag binds to β-catenin and acts as an effective inhibitor of the Wnt/β-catenin signaling pathway, thereby exhibiting potential as a treatment option for CRC.

Evidence demonstrates that the stable mobility of tumor cells obtained through EMT during the initiation process drives cell migration and metastasis [33]. The administration of 26ag reduced the migration and invasion capabilities of CRC cells associated with EMT inhibition. HCT-116 cells treated with 26ag up-regulated E-cadherin protein and mRNA expression; E-cadherin is an epithelial marker. Meanwhile, two mesenchymal markers, specifically N-cadherin and vimentin, were reduced. Moreover, 26ag also decreased the protein expression of Snail and Slug. Snail and Slug are the upstream transcription inhibitors of EMT and participate in tumor EMT by inhibiting the expression and activity of E-cadherin. Based on observations made during this study, it was suggested that 26ag enhances cell adhesion and the intracellular EMT process to inhibit the invasion and migration in HCT-116 cells.

Considering that Wnt/β-catenin is an important signal pathway regulating EMT and cell apoptosis, further experiments should investigate the underlying mechanism of 26ag on Wnt/β-catenin signaling. Our current results indicate that, predominantly, β-catenin was down-regulated after 26ag treatment. At the same time, according to immunofluorescence staining, translocation of β-catenin to the nucleus is inhibited after treatment with 26ag. Degradation of the APC/Axin/GSK-3β complex can directly affect the nuclear translocation of β-catenin [34]. 26ag significantly down-regulated the expression of GSK-3β and down-regulated the levels of the downstream TCF4. A significant marker that the Wnt signaling pathway is activated is that β-catenin stability in the nucleus decreases and it accumulates [35]. In addition, an interaction between 26ag and β-catenin was found through computer simulations of molecular docking, strongly supporting the notion that 26ag inhibits the Wnt/β-catenin pathway through interacting with β-catenin. It will be further necessary to use relevant experimental methods to study by what intermolecular interaction 26ag binds to the pocket of the β-catenin domain; for instance, a pull-down assay using purified recombinant β-catenin protein and generating co-crystal structures may be beneficial.

In summary, our study suggests that the small molecule compound 26ag shows promise for further development as an effective new inhibitor of the Wnt/β-catenin pathway for antagonizing CRC cell proliferation and migration. Although 26ag has a potential therapeutic window with IC_50_ of 3.6–5.5 µM for CRC cancer cells and 10.5 μM for non-cancerous L-O2 cells, developing it into CRC therapeutics by targeting Wnt/β-catenin needs to be cautiously performed. In the meantime, it is extremely necessary to conduct further animal studies.

## Figures and Tables

**Table 1:** The primers used in this study.

Gene	Primer Sequence
E-cadherin	forward primer:5′CGAGAGCTACACGTTCACGG-3′
	reverse primer: 5′-GGGTGTCGAGGGAAAAATAGG-3′
N-cadherin	forward primer: 5′-CCTTTCAAACACAGCCACGG-3′
	reverse primer: 5′-TGTTTGGGTCGGTCTGGATG-3′
vimentin	forward primer: 5′-GACGCCATCAACACCGAGTT-3′
	reverse primer: 5′-CTTTGTCGTTGGTTAGCTGGT-3′
Bax	forward primer:5′-TCAACTGGGGCCGGGTTGTC-3′
	reverse primer: 5′-CCTGGTCTTGGATCCAGCC-3′
Bcl-2	forward primer:5′-ATCGCTCTGTGGATGACTGAGTAC-3′
	reverse primer: 5′-AGAGACAGCCAGGAAAATCAAAC-3′
GAPDH	forward primer: 5′-CATCAAGAAGGTGGTGAAGCAGG-3′
	reverse primer: 5′-TCAAAGGTGGAGGAGTGGTGTCGC-3′

## Data Availability

The data presented in this study are available on request from the corresponding author.
